# Stiffened Hands in a Diabetic Patient: Diabetic Sclerodactyly

**DOI:** 10.1210/jcemcr/luac003

**Published:** 2022-11-29

**Authors:** Kavinga Gamage, Uditha Bulugahapitiya

**Affiliations:** Diabetes and Endocrinology Unit, National Hospital of Sri Lanka, Colombo 10, 01000, Sri Lanka; Diabetes and Endocrinology Unit, National Hospital of Sri Lanka, Colombo 10, 01000, Sri Lanka

**Keywords:** diabetic sclerodactyly, limited joint mobility

## Figure Legend

**Figure:**
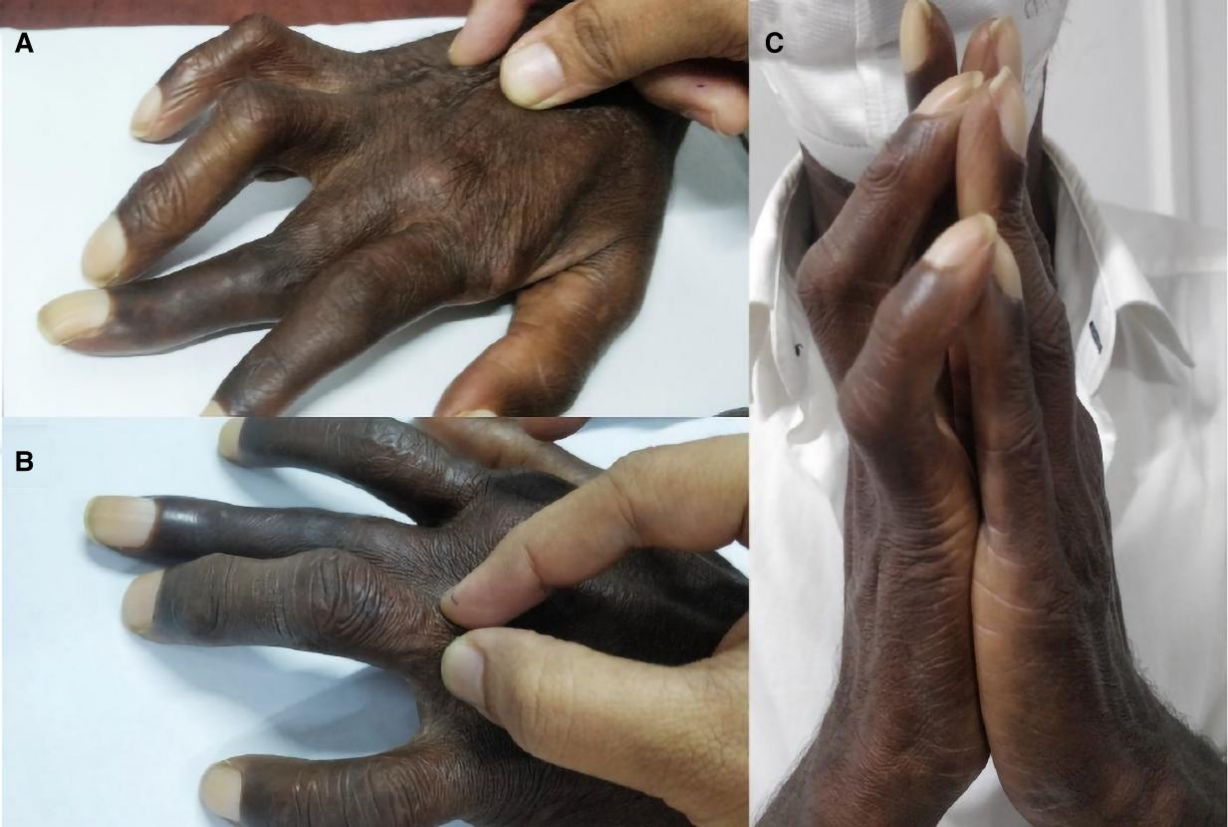


A 60-year-old male mechanic with type 2 diabetes for 10 years (glycated hemoglobin A_1c_ 9.8%) presented with limited mobility of bilateral fingers with progressive deformity, over 6 months. He had preexisting deformity of the second and the third digits of the right hand following a traumatic injury. There was no history suggestive of inflammatory arthritis or Raynaud phenomenon. He was a smoker, and consumed alcohol occasionally. Examination revealed tight skin over the dorsum of the hands with positive pinch sign, extending up to the distal forearm. Skin elsewhere was normal, including the face. Metacarpophalangeal and interphalangeal joint movements were markedly reduced bilaterally. Tapering of the fingers, cold extremities, telangiectasia, calcinosis cutis, speckled leukoderma, and ulcerations were absent. Nail fold capillaroscopy confirmed the absence of abnormal capillary architecture. Further examination revealed the presence of peripheral arterial disease. Inflammatory markers were normal. Diabetic sclerodactyly clinically imitates scleroderma by the presence of thickened skin but lacks other typical features of scleroderma, which is useful in differentiation (1). When severe it can affect the joint mobility. The presence of diabetic sclerodactyly may be associated with increased risk of macrovascular and microvascular complications (2).
